# New Trends in SARS-CoV-2 Variants and Vaccines

**DOI:** 10.3390/vaccines13030265

**Published:** 2025-03-03

**Authors:** Seik-Soon Khor

**Affiliations:** Singapore Centre for Environmental Life Sciences Engineering, Nanyang Technological University, Singapore 637551, Singapore; seiksoon@gmail.com

## 1. Introduction

The COVID-19 pandemic has significantly impacted global health, with the continued emergence of new SARS-CoV-2 variants presenting substantial challenges in terms of both vaccine efficacy and public health strategies. The Special Issue “New Trends in SARS-CoV-2 Variants and Vaccines” showcases the progress in understanding the virus’s structural evolution and vaccine-induced immunity and the introduction of innovative vaccination strategies. This editorial summarizes the main findings of the studies featured in this Special Issue.

## 2. Structural and Evolutionary Insights into SARS-CoV-2 Variants

Several studies in this Special Issue focused on the structural analysis of SARS-CoV-2 variants and their implications for the SARS-CoV-2 immune escape mechanism. Gerardi et al. [1] conducted a deep structural analysis of Omicron sub-variants and identified critical receptor-binding domain (RBD) mutations that enhanced viral binding affinity, contributing to immune escape. On the other hand, Olukitibi et al. [2] conducted a review on the importance of SARS-CoV-2 Spike (S) protein subunits for future vaccine development. In addition, Zhang et al. [3] reported the viral characteristics of the SARS-CoV-2 BA.2.87.1 lineage. These studies emphasized the rapid evolution of the SARS-CoV-2 virus and the challenges encountered when designing effective vaccines against emerging variants.

## 3. Vaccine-Induced Immunity and Booster Strategies

Previous studies have emphasized the importance of COVID-19 booster vaccinations in maintaining immunity against SARS-CoV-2. Meurisse et al. [4] investigated the effectiveness of homologous (mRNA-mRNA) and heterologous (viral vector–mRNA) prime–boost vaccination strategies, finding that mRNA booster doses significantly reduced the risk of severe disease and hospitalization. However, no differences were observed between homologous and heterologous prime–boost vaccination strategies. These findings highlight the critical role of mRNA-based booster doses in sustaining vaccine-induced immunity.

## 4. Alternative Vaccination Approaches: Intranasal and Dual-Antigen Vaccinations

Beyond traditional intramuscular vaccination, novel, effective, and less intrusive approaches, such as mucosal vaccines, have been explored. Ouyang et al. [5] investigated a mucosal vaccine candidate utilizing an rVSV-based bivalent vaccine against the SARS-CoV-2 delta variant. This vaccine demonstrated strong mucosal and systemic immune responses in preclinical models, suggesting its potential to enhance protection against the intranasal transmission route. Chiuppesi et al. [6] found that patients who have undergone hematopoietic cell transplantation (HCT) and those treated with chimeric antigen receptor (CAR)-T cell therapy are more susceptible to SARS-CoV-2 infection and produce a weaker immune response following COVID-19 vaccination. A new vaccine, COH04S1, co-expresses the spike (S) and nucleocapsid (N) antigens of SARS-CoV-2. Vaccination with COH04S1 resulted in heightened levels of S- and N-specific binding antibodies in these patients.

## 5. Strategies for Vaccination in Individuals with Compromised Immune Systems

Sripongpun et al. [7] studied the immune response to various vaccination regimens in patients with cirrhosis. They found that heterologous vaccination strategies offered better immunity than homologous regimens, especially when patients were boosted with mRNA vaccines. These findings emphasize the importance of developing diverse vaccine approaches tailored to different groups of patients.

## 6. Epidemiological and Modeling Studies on Vaccine Effectiveness

In addition to the immunological studies above, an epidemiological study can provide valuable insights into community vaccination strategies. A study conducted in Thailand [8] examined the seroprevalence of SARS-CoV-2 among unvaccinated individuals following the Delta variant outbreak. The results revealed low natural immunity levels in different regions of Thailand, highlighting the need for targeted vaccination efforts.

## 7. Conclusions

This Special Issue comprises studies that enhance our understanding of the evolution of SARS-CoV-2 variants, vaccine-induced immunity, vaccination strategies, and innovative vaccination approaches. Analyses of the structural characteristics and subtypes of SARS-CoV-2 reveal the mechanisms behind immune escape and immunogenicity and booster studies emphasize the importance of maintaining robust immune responses. Additionally, alternative vaccination strategies and real-world epidemiological data provide critical insights for optimizing vaccine deployment. As the COVID-19 pandemic evolves, these findings offer valuable guidance for shaping future vaccination policies and improving global preparedness against emerging SARS-CoV-2 variants.

## References

[1] Gerardi V., Rohaim M.A., Naggar R.F.E., Atasoy M.O., Munir M. (2023). Deep Structural Analysis of Myriads of Omicron Sub-Variants Revealed Hotspot for Vaccine Escape Immunity. Vaccines.

[2] Olukitibi T.A., Ao Z., Warner B., Unat R., Kobasa D., Yao X. (2023). Significance of Conserved Regions in Coronavirus Spike Protein for Developing a Novel Vaccine against SARS-CoV-2 Infection. Vaccines.

[3] Zhang L., Dopfer-Jablonka A., Nehlmeier I., Kempf A., Graichen L., Calderón Hampel N., Cossmann A., Stankov M.V., Morillas Ramos G., Schulz S.R. (2024). Virological Traits of the SARS-CoV-2 BA.2.87.1 Lineage. Vaccines.

[4] Meurisse M., Catteau L., van Loenhout J.A.F., Braeye T., De Mot L., Serrien B., Blot K., Cauët E., Van Oyen H., Cuypers L. (2023). Homologous and Heterologous Prime-Boost Vaccination: Impact on Clinical Severity of SARS-CoV-2 Omicron Infection among Hospitalized COVID-19 Patients in Belgium. Vaccines.

[5] Ouyang M.J., Ao Z., Olukitibi T.A., Lawrynuik P., Shieh C., Kung S.K.P., Fowke K.R., Kobasa D., Yao X. (2023). Oral Immunization with rVSV Bivalent Vaccine Elicits Protective Immune Responses, Including ADCC, against Both SARS-CoV-2 and Influenza A Viruses. Vaccines.

[6] Chiuppesi F., Ortega-Francisco S., Gutierrez M.-A., Li J., Ly M., Faircloth K., Mack-Onyeike J., La Rosa C., Thomas S., Zhou Q. (2023). Stimulation of Potent Humoral and Cellular Immunity via Synthetic Dual-Antigen MVA-Based COVID-19 Vaccine COH04S1 in Cancer Patients Post Hematopoietic Cell Transplantation and Cellular Therapy. Vaccines.

[7] Sripongpun P., Pinpathomrat N., Sophonmanee R., Ongarj J., Seepathomnarong P., Seeyankem B., Chamroonkul N., Piratvisuth T., Kaewdech, A., on behalf of the Cirrhosis-COVID-19 Vaccine Study Group (2023). Heterologous COVID-19 Vaccination and Booster with mRNA Vaccine Provide Enhanced Immune Response in Patients with Cirrhosis: A Prospective Observational Study. Vaccines.

[8] Mahasirimongkol S., Uppapong B., Puangtubtim W., Dhepakson P., Panyajai P., Thawong N., Pinyosukhee N., Rojanawiwat A., Wichukchinda N., Soonthorncharttrawat S. (2022). SARS-CoV-2 Seroprevalence in Unvaccinated Adults in Thailand in November 2021. Vaccines.

